# Supplementary material for: Assessing Metabolic Markers in Glioblastoma Using Machine Learning: A Systematic Review
Source: Metabolites. 2023 Jan 21;13(2):161. doi: 10.3390/metabo13020161 (PMC9958885; doi:10.3390/metabo13020161)
Supplement: Supplementary file 1 [file metabolites-13-00161-s001.zip › metabolites-2153726-supplementary.pdf]

Supplementary Materials

Supplementary Materials S1: Search String

PubMed
(((Metabolism[MeSH Terms]) OR (biomarkers[MeSH Terms])) **AND** ((Glioblastoma[Title/Abstract]) OR (glioblastoma[MeSH Terms]))) **AND** (((((artificial intelligence[MeSH Terms]) OR ("Artificial Intelligence"[Title/Abstract])) OR ("Machine Learning"[Title/Abstract])) OR ("Deep Learning"[Title/Abstract])) OR ("Predictive model"[Title/Abstract]))

Embase
('metabolism':ti,ab OR 'biological marker':ti,ab) AND 'glioblastoma' AND ('artificial intelligence':ti,ab OR 'machine learning':ti,ab OR 'deep learning':ti,ab OR 'predictive model':ti,ab)

Web of Science
((“metabolism” OR “biomarkers”) AND “glioblastoma” AND (“artificial intelligence” OR “machine learning” OR “deep learning” OR “predictive model”)).ti,ab.

Ovid
((“metabolism” OR “biological marker”) AND “glioblastoma” AND (“artificial intelligence” OR “machine learning” OR “deep learning” OR “predictive model”)).ti,ab.

Cochrane
("glioblastoma"):ti,ab AND ("machine learning" OR "deep learning" OR "artificial intelligence" OR "predictive model"):ti,ab AND ("metabolism" OR "biomarkers"):ti,ab

Table S1: Risk of Bias Assessment

STUDY	TYPE	LEVEL OF EVIDENCE	RISK OF BIAS					APPLICABILITY CONCERNS			
			PATIENT SELECTION	INDEX TEST	REFERENCE STANDARD	FLOW AND TIMING	ANALYSIS	PATIENT SELECTION	INDEX TEST	REFERENCE STANDARD	FLOW AND TIMING
Ishwar (2022)	Diagnostic	II	UNSURE	UNSURE	LOW	UNSURE		UNSURE	LOW	LOW	
McInerney (2022)	Both	II	HIGH	LOW	HIGH	LOW	LOW	LOW	LOW	LOW	LOW
Firdous (2021)	Diagnostic	I	HIGH	LOW	UNSURE	UNSURE		LOW	LOW	LOW	
Jia (2021)	Prognostic	II	HIGH	UNSURE	UNSURE	LOW	UNSURE	LOW	LOW	LOW	LOW
Kaluzinska (2021)	Prognostic	II	UNSURE	UNSURE	UNSURE	HIGH	UNSURE	LOW	LOW	LOW	LOW
He (2020)	Prognostic	II	HIGH	UNSURE	UNSURE	UNSURE	UNSURE	LOW	LOW	UNSURE	UNSURE
Zeng (2019)	Prognostic	II	UNSURE	LOW	HIGH	UNSURE	UNSURE	LOW	LOW	LOW	UNSURE
Hao (2018)	Prognostic	II	HIGH	HIGH	HIGH	UNSURE	HIGH	LOW	LOW	LOW	HIGH
Shu (2018)	Prognostic	II	HIGH	UNSURE	UNSURE	HIGH	HIGH	LOW	LOW	UNSURE	UNSURE
Gollapalli (2012)	Diagnostic	II	HIGH	HIGH	UNSURE	UNSURE		LOW	UNSURE	LOW	

Risk of bias assessment based on Quality Assessment of Diagnostic Accuracy Studies 2 (QUADAS-2) for diagnostic studies and the Quality Assessment of Prognostic Accuracy Studies (QUAPAS) for prognostic studies.
